# Primary aldosteronism: adrenalectomy could save more lives

**DOI:** 10.3389/fendo.2025.1737160

**Published:** 2025-12-08

**Authors:** Tobias Carling, Fabio R. Faucz

**Affiliations:** 1 Carling Adrenal Center, Tampa, FL, United States; 2 Hospital for Endocrine Surgery Research Center, HCA Healthcare Research Institute, HCA Florida Healthcare, Tampa, FL, United States

**Keywords:** adrenalectomy, primary aldosteronism, aldosterone, mortality, underdiagnosis, Conn syndrome, hyperaldosteronism

## Abstract

Primary aldosteronism (PA), a leading cause of secondary hypertension, remains profoundly underdiagnosed and, contributes to vast preventable cardiovascular morbidity and mortality worldwide. Affecting 4.5–20% of patients with high blood pressure, PA drives excess aldosterone production, substantially elevating risks of heart failure, stroke, atrial fibrillation, and renal damage, with untreated cases facing 10–20% higher mortality rates over 5–10 years compared to essential hypertension. Adrenalectomy for unilateral PA driven by somatic mutations (e.g., *KCNJ5*) offers markedly superior outcomes over medical therapy, yielding an absolute risk reduction in all-cause mortality that rivals or exceeds many major cardiovascular surgeries and greatly improves quality of life by resolving hypertension, hypokalemia, and associated symptoms. Advanced surgical approaches such as mini back scope adrenalectomy (posterior retroperitoneoscopic) minimize risks and enable precise, function-preserving surgery. Yet, current surgical rates, ranging from 1.2 to 8.4 per million across countries like Sweden, Taiwan, France, UK, Germany, and USA, represent less than 4% of the optimal rate of 212 operations/million. Achieving optimal adrenalectomy rates requires increasing surgical capacity 25–170-fold, potentially saving 5,627 lives annually in the USA alone. Disparities arise from diagnostic barriers, including underutilization of aldosterone-to-renin ratio screening, complex confirmatory testing, and limited access to adrenal vein sampling and high-volume adrenal surgeons. This perspective highlights PA’s severe burden, compares adrenalectomy’s efficacy to major cardiac and vascular operations, and examines international caseload variations linked to research infrastructure, PA guidelines, and access to high-volume centers. We strongly urge enhanced PA screening, improved case detection, diagnostic workflows, and expanded surgical access to avert this silent epidemic.

## Introduction

Primary aldosteronism (PA), a prevalent yet underrecognized cause of secondary hypertension, contributes significantly to preventable cardiovascular-related deaths (1–5). The disease is due to unifocal (aldosterone-producing adenoma; APA) or multifocal tumors, or more rarely aldosterone-producing diffuse hyperplasia (APDH) (6). Despite the proven efficacy of adrenalectomy in curing this condition, its application remains limited (7, 8). Optimizing surgical intervention rates for PA in nations such as Sweden, Taiwan, France, the UK, Germany, and the USA could prevent thousands of premature deaths, achieving a life-saving impact similar to some major cardiovascular operations. In other international regions, where PA adrenalectomy rates are likely even lower, millions remain undiagnosed and untreated, fueling a silent yet devastating public health crisis. We evaluated the mortality reduction achieved through adrenalectomy for PA compared to major cardiac and vascular surgeries, alongside their impact on quality of life (**Table 1**), and estimated current versus optimal global PA adrenalectomy rates (**Table 2**). This perspective examines the multifaceted burden of PA, its diagnostic challenges, the superior efficacy of surgical intervention, and actionable strategies to shift to a proactive, life-saving paradigm for PA management.

**Table 1:** Mortality impact of adrenalectomy for primary aldosteronism (PA) compared to commonly employed major cardiovascular operations and the effect on quality of life.

Procedure	NNT	ARR (%)	Mortality notes	Time horizon	QoL effect*	Average age at operation	Reference
TAVR (severe aortic stenosis)	4–10	10–26.8	High-risk: ARR ~26.8% vs. BMT; reduces CV mortality	1–5	Improved	~80–84	(29)
**Adrenalectomy (PA)**	**13**	**7.9**	**Reduced CV/stroke mortality vs. BMT**	**6**	**Improved**	**~50–55**	(1)
Elective repair, AAA (5 cm)	10–20	5–10	Annual rupture risk ~1–2%	10	Minimal	~70–75	(30)
Elective CABG (stable CAD)	25	4	Reduced all-cause mortality vs. BMT; greater benefit in left main (NNT 8) or triple-vessel disease (NNT 25); periprocedural mortality ~1–2%	10	Improved (angina relief, but cognitive decline in 20–30%)	~65–70	(32)
Elective CEA (asymptomatic carotid stenosis)	37	2.7	Reduced disabling/fatal stroke (half of stroke risk reduction); periprocedural stroke/death ~3%	10	Minimal	~70–72	(31)

Adrenalectomy for primary aldosteronism (PA) is in bold. NNT, Number needed to treat to prevent one death (or disabling/fatal stroke for CEA) vs. surveillance/best medical therapy (BMT); ARR. Absolute risk reduction; Time horizon and average age at operation are in years; QoL, Quality of life; TAVR, Transcatheter aortic valve replacement; CV, Cardiovascular; AAA, Abdominal aortic aneurysm; CABG, Coronary artery bypass grafting; CAD, Coronary artery disease; CEA, Carotid endarterectomy. *QoL assumes stable/asymptomatic baseline; complications may reduce QoL.

**Table 2:** Current annual rates of adrenalectomy for primary aldosteronism (PA) and optimal rates (212 per million population, in bold) in various countries.

Country (population)	PA adrenalectomies total	PA adrenalectomies per million	% PA adrenalectomies	*Optimal PA adrenalectomies	Reference
Sweden (10.7M)	~88	~8.4	~39%	**~2,259**	(36)
Taiwan (23.1M)	~90	~3.9	~20%	**~4,900**	(24)
France (66.6M)	~83	~1.2	~5.0%	**~14,130**	(37)
UK (69.5M)	~160	~2.4	~20%	**~14,744**	(38)
Germany (84.1M)	~500	~6.0	~23%	**~17,823**	(43)
USA (347.3M)	~492	~1.4	~8.2%	**~73,622**	(39)

*Optimal PA adrenalectomies rates (in bold) assume full case detection of unilateral primary aldosteronism (PA; 212/million; 35% of all PA). PA adrenalectomy rates are as a % of all adrenalectomy cases (as standalone procedure, and not as part of other operations, e.g. radical nephrectomy). The estimates are based on data from Socialstyrelsen (Sweden), National Health Insurance (NHI) (Taiwan), ATIH (France), Hospital Episode Statistics (UK), DRG data (Germany) and Nationwide Inpatient Sample and NSQIP (USA), and supplemented with data from publications using the Scandinavian Quality Register for Thyroid, Parathyroid and Adrenal Surgery (SQRTPA; Sweden), Taiwan Primary Aldosteronism Investigation group TAIPAI (Taiwan), Eurocrine/AFCE (France), UK Registry of Endocrine and Thyroid Surgery (UKRETS; UK), Eurocrine (Germany), and Collaborative Endocrine Surgery Quality Improvement Program (CESQIP; USA) and, personal communications.

## Primary aldosteronism as driver of morbidity and mortality

PA is increasingly acknowledged as a significant health challenge due to its high prevalence and profound cardiovascular, renal, and metabolic consequences (1–4). Affecting an estimated 4.5–20% of individuals with hypertension, equivalent to up to 12,100 cases per million, PA is characterized by excessive aldosterone production, which significantly elevates mortality risk by 10–20% over 5–10 years compared to essential hypertension (3–5). Predominantly impacting working-age adults, PA imposes substantial societal burdens through chronic morbidity, reduced workforce productivity, and escalating healthcare costs. Without intervention, patients face markedly elevated risks, including a 2.5–4-fold increase in stroke, a 2.6–6.5-fold increase in non-fatal myocardial infarction, and a 3.2–12-fold increase in atrial fibrillation compared to those with essential hypertension (4, 9–11). These data underscore the urgent need for enhanced screening, diagnosis, and management strategies to mitigate the far-reaching impacts of this condition. Excess aldosterone causes extensive target organ damage beyond blood pressure elevation. In the cardiovascular system, it promotes myocardial fibrosis, left ventricular hypertrophy, and endothelial dysfunction, leading to heart failure, arrhythmias, and accelerated atherosclerosis risk (2, 4, 9, 12). In the kidneys, aldosterone induces glomerular hyperfiltration, proteinuria, and progressive renal fibrosis, contributing to chronic kidney disease and end-stage renal failure (13, 14). Additionally, PA exacerbates metabolic disturbances, including insulin resistance, dyslipidemia, and vascular inflammation, amplifying systemic damage and cardiovascular risk (3, 4, 13). Concomitant hypercortisolism in PA is increasingly recognized as a major contributor to morbidity, as well. Co-secretion of cortisol in APAs or bilateral PA, reported in up to 20–30% of PA cases, compounds cardiovascular and metabolic risks by promoting visceral obesity, glucose intolerance, and osteoporosis, while exacerbating hypertension and hypokalemia (15, 16). This dual hormonal excess intensifies end-organ damage, and worsens quality of life, underscoring the need for comprehensive hormonal screening in PA patients to guide targeted surgical or medical interventions (15, 16).

Frequently mistaken for essential hypertension, PA escapes detection in millions, sustaining a hidden epidemic. Innovative procedures like mini back scope adrenalectomy (MBSA, also known as posterior retroperitoneoscopic), completed in under 20 minutes by experienced surgeons, can restore hormonal balance, resolve hypertension, and reduce cardiovascular mortality, with very low risks of complications (7, 17, 18). Approximately 35% of PA cases stem from a unilateral APA curable by surgery, although data from different centers range between 25-70% dependent on referral patterns, and stringency of diagnostic tools such as confirmatory testing and AVS methodology (19).

Unfortunately, many patients suffer prolonged poor blood pressure control, either undiagnosed, or managed by ever-increasing pharmacological therapies without identifying the root cause and a severely reduced quality of life, unaware that a low-risk operation could prevent severe complications (1, 7, 20, 21). Recent meta-analyses confirm surgery’s edge in reducing major adverse cardiovascular events, with quicker and more pronounced benefits than medical management (10, 20–22). A comprehensive nationwide study evaluated mortality in PA and the effect of adrenalectomy in 2,419 patients with PA and 24,187 controls with a follow up of 8.1 years. PA was associated with increased risk of all-cause mortality from cardiovascular disease and stroke. Patients with cardiovascular disease at diagnosis, patients treated with a low dose of a mineralocorticoid receptor antagonist (MRA), and untreated patients had excess mortality. Conversely, patients treated with unilateral adrenalectomy had a decreased mortality risk, which was similar to age- and sex-matched controls without PA, *i.e.* patients who underwent adrenalectomy remarkably returned to a baseline risk similar to the general population (23). Furthermore, genetic research reveals somatic mutations in aldosterone-producing adenomas, including: *KCNJ5* potassium channel mutations in 43-73% of cases, *CACNA1D* calcium channel mutations in 9-21%, *ATP1A1* sodium/potassium ATPase mutations in 5-8%, and *ATP2B3* plasma membrane calcium ATPase mutations in 1-3%. These mutations provide potential molecular targets for diagnostics and therapies as they disrupt ion homeostasis leading to autonomous aldosterone secretion. *KCNJ5* mutations, in particular, are linked to better post-adrenalectomy outcomes often with more complete hypertension remission due to distinct adenoma features (24–26). Bilateral PA, traditionally managed medically, is increasingly treated with staged, partial (function-preserving) adrenalectomy in select cases, negating the need for lifelong glucocorticoid replacement, particularly in patients with *ARMC5* germline mutations that promote bilateral macronodular hyperplasia predisposing to bilateral disease (7, 17, 27, 28).

Admittedly, adrenalectomy’s risk reduction in PA relies on relatively limited data, hindering definitive answers to two key management questions: the extent of PA’s contribution to morbidity and mortality, and its reversibility by adrenalectomy. Current literature is restricted by incomplete data on PA’s biochemical severity and duration before surgery, difficulties in accounting for common comorbidities like essential hypertension, chronic kidney disease, obesity, and diabetes, dependence on retrospective cohort studies, and meta-analyses of mainly observational data. Still, growing evidence supports substantial morbidity and mortality benefits following adrenalectomy (1–4).

## The efficacy of adrenalectomy in PA

We evaluated the mortality reduction achieved through adrenalectomy for PA, comparing its efficacy to major cardiovascular surgeries and their effects on quality of life (**Table 1**). The mortality impact of adrenalectomy for PA was estimated using previously published data (1, 2, 20, 23), and then compared to commonly employed major cardiovascular operations. The absolute risk reduction (ARR) and a number needed to treat (NNT, i.e. the number of procedures performed to save one life) were calculated for each surgical intervention. Adrenalectomy for PA achieves a 7.9% ARR in cardiovascular mortality over six years, with an NTT of 13 (1, 2, 20). This significant ARR, strongly derived from several meta-analyses and nationwide retrospective studies of appropriately selected surgical cohorts for PA, indicates the profound benefit of adrenalectomy (1, 2, 20, 23), which is comparable only to transcatheter aortic valve replacement (TAVR) for severe aortic stenosis (29). While this ARR represents a powerful health gain, it is important to note that it is based on studies of patients deemed eligible for surgery. Consequently, its applicability to older individuals or those with significant comorbidities may be reduced, and absolute benefits may be smaller in low-risk or elderly populations, especially since age-specific variations in ARR for adrenalectomy in PA are not yet widely available in the literature (23). Adrenalectomy for PA ranks above elective repair of a 5 cm abdominal aortic aneurysm (AAA), where untreated rupture poses significant mortality risks (30), as well as elective coronary artery bypass grafting (CABG) for stable coronary artery disease and carotid endarterectomy (CEA) for asymptomatic carotid artery stenosis for reduction in stroke risk (31, 32). Furthermore, PA primarily affects younger, working-age adults, with an average age of 50–55 years at the time of adrenalectomy (1, 7, 23). This is significantly younger than the average age for the listed major cardiovascular surgeries, which typically occur in a patient’s 70s or 80s (**Table 1**). Thus, treating PA surgically can lead to several decades of improved lifespan, quality of life, reduced productivity losses, and healthcare costs.

Beyond survival, adrenalectomy significantly improves quality of life by correcting hormonal imbalances, easing hypertension, muscle cramps, headaches, obesity, sleep apnea, fatigue, and anxiety, with minimal complications in skilled hands (7, 24, 33). Minimally invasive approaches, including partial (function-preserving) adrenalectomy, enhance safety and preserve adrenal function, particularly important in bilateral and/or multifocal PA (7, 17, 18). Long-term studies show sustained reductions in cardiovascular events post-surgery, with unilateral PA patients achieving better outcomes than medically treated counterparts, notably in those with *ATP1A1* mutations tied to more severe preoperative profiles (1, 10, 20, 23, 34). In bilateral PA, international cohorts demonstrate feasibility of less-than total adrenalectomy with biochemical cure rates ranging from 65-85%, while maintaining a very low risk of postoperative hypocortisolism, negating the need for glucocorticoid replacement therapy (7, 27). Similar to PA, adrenalectomy seems underused also in various types of adrenal hypercortisolism and pheochromocytoma (35).

## Global potential for surgery in PA

We estimated the current and optimal rates of PA adrenalectomy across various countries using data from national registries, administrative datasets, peer-reviewed studies and personal communications (**Table 2**). Briefly, the estimates are based on data from Socialstyrelsen (Sweden), National Health Insurance (NHI) (Taiwan), ATIH (France), Hospital Episode Statistics (UK), DRG data (Germany) and Nationwide Inpatient Sample and NSQIP (USA), and supplemented with data from publications using the Scandinavian Quality Register for Thyroid, Parathyroid and Adrenal Surgery (SQRTPA; Sweden), Taiwan Primary Aldosteronism Investigation group TAIPAI (Taiwan), Eurocrine/AFCE (France), UK Registry of Endocrine and Thyroid Surgery (UKRETS; UK), Eurocrine (Germany), and Collaborative Endocrine Surgery Quality Improvement Program (CESQIP; USA) and, personal communications. Current PA adrenalectomy rates vary widely across Sweden, Taiwan, France, the UK, Germany, and the USA. For example, France performs approximately 83 adrenalectomies per year (1.2 per million), similar to Sweden’s 88 cases (8.4 per million), despite France’s population being 6.2 times larger (36, 37). Similarly, the proportion of adrenal operation for PA as a percentage of all stand-alone adrenalectomies (and not as part of other operations, e.g. radical nephrectomy) was highest in Sweden (~39%) and lowest in France and the USA (~5.0%, and 8.2%), respectively. However, assuming 35% of PA cases (i.e. curable unilateral PA) were properly referred for surgery, the PA adrenalectomy rates in all analyzed countries fall dramatically short of the optimal (38, 39). This assumption, reflecting the proportion of diagnosed PA cases amenable to surgical cure (as opposed to all undiagnosed PA patients), highlights a dramatic shortfall in current adrenalectomy rates. This shortfall translates to ~73,130 missed procedures annually in the USA alone, causing ~5,627 deaths per ARR estimates (1, 39). It is crucial to acknowledge that these estimations, which inherently represent upper-bound projections assuming complete case detection and surgical candidacy, while based on diagnosed unilateral PA and representing an ideal scenario, may overstate the true number of surgical candidates. This is because the actual fraction of surgically eligible patients in unselected hypertensive populations remains uncertain and is likely lower after accounting for age, comorbidities, and patient preference. However, given the vast underdiagnosis, the potential for increasing lives saved remains substantial. Conversely, it may also underestimate such numbers as in some populations the prevalence of surgically curable PA may actually be higher. To reach optimal rates of PA adrenalectomies, each analyzed country would need to increase its current number of adrenal operations for PA by a factor of ~25 to 170 times. In regions with less developed diagnostic and surgical infrastructure, PA adrenalectomy rates are surely even lower, leaving millions of patients undiagnosed and without optimal treatment globally (4, 19).

Country-specific rates reveal disparities tied to healthcare and screening variations. Sweden leads with both the highest rate of PA adrenalectomies both per million, and as a percentage of all adrenalectomies, aided by universal healthcare coverage, national registries like SQRTPA, strong PA research, and rapid implementation of novel science into clinical practice (23, 33, 36, 40). Taiwan at 3.9 PA adrenalectomies per million, benefits from robust research via the Taiwan Primary Aldosteronism Investigation (TAIPAI) group, capturing ~80% of national cases, and national clinical practice guidelines (24, 41). Taiwanese scientists have been early proponents of both partial (function-preserving) adrenalectomy and adrenalectomy for select cases of bilateral PA, reflecting a proactive, and evidence-based approach (27, 41, 42). Germany, with a comparably high rate of PA adrenalectomies, benefits from high-volume centers and Eurocrine registries, similar to Sweden and Taiwan (17, 43). In contrast, the USA, the UK and France trail, with abysmally low PA case detection, scant patients appropriately referred for surgery, and very low percentage PA adrenalectomy rates (37–39). These disparities reflect variations in research ecosystems, national registries, clinical guidelines, and access to high-volume centers, which drive higher caseloads in countries like Sweden, Taiwan, and Germany.

## Diagnostic challenges

The underuse of adrenalectomy for PA arises from substantial diagnostic challenges. The aldosterone-to-renin ratio test, essential for PA screening, is performed in fewer than 2-5% of hypertensive patients, despite guideline recommendations (19, 40, 44). The July 2025 Endocrine Society guidelines now advocate for universal biochemical screening for PA in all hypertensive patients, signaling a major shift toward detecting this overlooked condition (19, 44). Yet, confirmatory tests like saline loading or adrenal vein sampling (AVS) are complex and resource-intensive, delaying referrals in strained healthcare systems (7, 45, 46). AVS, essential for determining lateralization in unilateral PA, has limitations including technical failure rates, variable interpretation and inadequate availability, prompting calls for imaging-based alternatives in select cases (3, 45, 46). The lack of endocrinology expertise among primary care providers and insufficient specialists exacerbates the issue. Unlike well-established screening for other conditions (e.g. AAA and certain cancers), PA lacks routine hormone checks in annual physician visits or national monitoring similar to those via cancer registries.

## Discussion and path forward

Tackling PA underdiagnosis requires a comprehensive diagnostic and therapeutic overhaul. Routine screening for PA, measuring aldosterone, renin, and potassium, should be standard in primary care for all hypertensive patients, as advocated by the new 2025 Endocrine Society PA guidelines, potentially uncovering thousands of surgical candidates (19, 44). Such screening could yield substantial healthcare savings by preventing costly cardiovascular events. Extending universal screening to high-risk groups, such as those with resistant hypertension, hypokalemia, atrial fibrillation, sleep apnea, early-onset hypertension, or a family history of cardiovascular events, could further enhance case detection (3, 4). Improved patient stratification into mild, moderate, and severe disease categories, as outlined in recent expert consensus, allows for tailored management (47). For instance, patients with moderate to severe PA can often bypass confirmatory testing and AVS, proceeding directly to surgery when imaging is unequivocal (47). Conversely, mild cases can often be managed by pharmacotherapy alone avoiding AVS and surgery, which has a lower yield in such cases, unless progressive PA occurs. The advent of functional positron emission tomography (PET) scanning for PA, offers a non-invasive alternative for lateralization, reducing the reliance on AVS, which has a 10–92% failure rate (largely dependent on the operator experience) and frequently limited availability (45, 46, 48–51). PET imaging further shows high sensitivity and specificity in subtyping PA, enabling faster and more accessible diagnosis, although it is not yet widely available (49, 51). Postoperative histopathological evaluation using the HISTOALDO consensus, incorporating CYP11B2 immunohistochemistry to map aldosterone-producing zones, is critical for accurate postoperative PA subtyping, guiding prognosis and follow-up care as well as determining whether additional contralateral function-preserving adrenalectomy is indicated (6, 7, 27). Such a surgical approach preserves adrenal tissue, minimizing the risk of adrenal insufficiency, even in the subset of patients requiring bilateral surgery (7, 17). Furthermore, expanding adrenalectomy to bilateral PA, through unilateral or function-preserving bilateral procedures, may offer better control than medical therapy in select patients, reducing long-term MRA-related complications like lack of libido, erectile dysfunction, gynecomastia, menstrual irregularities, and hyperkalemia. Admittedly, the long-term results in biochemical cure and effects of cardiovascular morbidity and mortality are currently unknown with this approach, but the risk of adrenal insufficiency seems very low (<1-2%) (7, 27). To address surgical capacity and disparities, establishing high-volume adrenal centers worldwide is critical to ensure equitable access to specialized care (7). For example, the Carling Adrenal Center, where one author (TC) performs approximately 130 PA adrenalectomies annually, ~1.6 times France’s national total, demonstrates the potential for rapid capacity expansion (37). Some propose using non-surgical approaches like radiofrequency ablation, especially for carefully selected patients with exclusively left-sided APAs who are inoperable or refuse surgery. However, such approaches provide no pathological data and will likely lead to unacceptably high rates of both biochemical persistence and recurrence of PA (52).

Streamlining diagnostics with using artificial intelligence and machine learning of clinical and biochemical data could potentially enhance scalability, while public campaigns could raise awareness and encourage testing for PA (53). Boosting primary care training in endocrinology and increasing specialist access are essential to bridge the diagnostic divide, particularly in resource-limited settings. Emerging therapies, such as aldosterone synthase inhibitors, offer promising non-surgical options for managing mild or bilateral PA without tumors, particularly for patients intolerant to MRAs, or those unsuitable for surgery, thereby expanding treatment options (53–55).

In conclusion, the ongoing underuse of adrenalectomy for PA claims preventable lives, making delay highly concerning, and thus, action to optimize case detection, diagnostic and treatment stratification and surgical rates is imperative. By emulating and enhancing the models of Sweden, Taiwan, and Germany, e.g. integrating research, registries, and high-volume centers, global healthcare systems can close disparities, prioritize PA, and realize adrenalectomy’s full potential in saving lives and improving quality of life.

## Data Availability

The original contributions presented in the study are included in the article/supplementary material. Further inquiries can be directed to the corresponding author.
